# Exploring False Warnings and Modalities for Emergency Vehicle Alerts and Their Impact on Driver Behavior

**DOI:** 10.1177/00187208261436744

**Published:** 2026-03-21

**Authors:** Kajsa Weibull, Marius Brudvik Norell, Birgitta Thorslund, Björn Lidestam

**Affiliations:** 1 25543 Swedish National Road and Transport Research Institute, Sweden; 2 4566 Department of Computer and Information Science, Linköping University, Sweden

**Keywords:** driver behavior, intelligent vehicle systems, warnings, warning systems, warning compliance

## Abstract

**Objective:**

This study explores the order effects in false alarms and the effect of warning modality when drivers receive an Emergency Vehicle Approaching (EVA) alert.

**Background:**

EVA alerts have the potential to decrease delays for emergency vehicles. However, false alarms and suboptimal warning designs can decrease warning effectiveness. Aligned with previous research, drivers should benefit from receiving an alert that is not visual.

**Method:**

A driving simulator study with 80 participants was conducted. During a 30-minute drive, the participants received three EVA alerts, one of which was false. One group received a false alert first, followed by a true alert. The other group had the opposite order. Both groups received another true alert before the experiment ended. Half of the drivers received auditory warnings, and the other half visual warnings.

**Results:**

Drivers who received auditory warnings moved over more quickly compared to drivers who received visual alerts. There was an order effect of false warnings, suggesting that drivers who had received a false warning before they received a true warning were slower at moving over, compared to drivers who received a true warning in their first interaction. However, when the drivers received one true and one false warning each, there was no order effect in the third interaction.

**Conclusion:**

Both alert modality and false warning effects are important when implementing EVA alerts.

**Application:**

This research should be used to understand the order effect of false warnings on in-car warnings. When designing in-car warnings, auditory warnings may be more effective.

## Introduction

The deployment and arrival of care through emergency vehicles are often the most time-consuming steps in the emergency response chain (The Swedish National Board of Social Affairs and Health, 2023). Emergency vehicle operators face highly demanding tasks in stressful situations due to the critical importance of time and unavoidable interactions with other road users (Burke et al., 2001; Hsiao et al., 2018; Koski & Sumanen, 2019).

The alerts used by emergency vehicles, such as sirens and warning lights, represent signals that may be hard for civilians to detect and localize (Caelli & Porter, 1980). As modern vehicles become increasingly soundproof, the effectiveness of today’s sirens decrease (Weibull et al., 2023a). If effective, warnings for emergency vehicles can increase safety and potentially decrease arrival times (Wiwekananda et al., 2020), benefiting society through more efficient healthcare (Lenné et al., 2008; Lidestam et al., 2020) and economic advantages (Emanuelsson et al., 2023).

An alternative to warning lights and sirens is the use of Emergency Vehicle Approaching (EVA) alerts (Lenné et al., 2008). EVA is an in-car warning system that encourages drivers to move over, providing additional time to do so in a safe manner. The EVA system has been demonstrated as effective in several studies (Lenné et al., 2018; Lidestam et al., 2020; Savolainen et al., 2010; Weibull, Lidestam, & Prytz, 2024). Previous studies with EVA warnings have explored different alert modalities. Namely, either visual (Savolainen et al., 2010), auditory (Weibull et al., 2025), or a combination of visual and auditory (Lenné et al., 2018; Lidestam et al., 2020; Payre & Diels, 2020; Weibull, Lidestam, & Prytz, 2024; Weibull, Kunclová, et al., 2024).

## Warning Modality

Successfully distributing a warning, in a car or in another setting, to a receiver can be difficult. There are multiple ways in which the warning can get lost along the way. The steps in warning communication are illustrated in the Communication-Human Information Processing (C-HIP) model (Wogalter, 2006). One of the steps in the model is the channel through which the warning is presented. One aspect is the warning modality. Visual alerts are physically anchored to their source and can use symbols, color, and text to communicate effectively (Edworthy & Adams, 1996). Because they are less intrusive than other types of alerts, they can also be easier to miss, especially if presented outside the user’s field of view. With no inherent attention directing mechanism beyond sudden appearance, their urgency must be conveyed visually, for example, through color intensity, contrast, or symbolism. The presence of hazard design elements, such as icons, could affect the receiver’s attention to the warning (Hendricks & Peres, 2023). In contrast to visual warnings, auditory warnings are not bound to a location. They extend beyond their source (Edworthy & Adams, 1996). Challenges include ensuring detectability and enabling localization, as sound does not offer the same spatial specificity as a visual cue. Effective auditory warnings require careful design choices, including appropriate volume, repetition, and distinctiveness from environmental sounds. Understanding the operational environment and aligning the design with perceptual and cognitive needs is essential to creating effective warnings (Edworthy & Adams, 1996).

The choice of warning modality could affect the drivers’ sense of urgency and the perceived level of annoyance (Baldwin & Lewis, 2014). It is therefore important that the warning is designed in such a way that it communicates a correct level of urgency (Dingus et al., 1997). Increasing the sound in an auditory alert could increase the sense of urgency, but it can also increase the level of driver annoyance (Baldwin, 2011).

An important aspect when deciding what warning modality to use is to consider the sensory input that the user is receiving in the environment when not receiving the warning. According to Multiple Resource Theory, mental resources are multiple in contrast to a single undifferentiated capacity (Wickens, 2008). Hence, different types of tasks and different combinations of the tasks will affect the available mental resources differently. For instance, it is easier to combine tasks that require different types of cognitive resources. It is easier to visually scan the environment while listening to the radio at the same time than to visually scan the environment and read at the same time. Driving is mainly a visual task (Vilchez et al., 2024); therefore, drivers should benefit from receiving an auditory EVA alert, rather than a visual one. In addition, several studies have found that an auditory warning leads to faster reaction times compared to visual warnings (Bella & Silvestri, 2017; Huang et al., 2024; Lylykangas et al., 2016). At the same time, it is important to consider possible perceptual issues in driving. For instance, if the driver is accompanied by noisy passengers, it could be difficult to hear the alert. In addition, the driver might have impaired hearing ability. Besides visual and auditory alerts, in-car warning systems with tactile feedback have been shown to increase urgency without increasing annoyance (Baldwin & Lewis, 2014).

Weibull, Lidestam, and Prytz (2024) examined the difference between visual and auditory modalities in an in-car EVA system. The auditory alert consisted of a voice (in Swedish) saying, “Warning! Emergency Vehicle! Please give way,” that was distributed through the car’s speakers. The visual alert was a dashboard sign stating “Emergency vehicle alert. Drive with caution.” There was no significant difference in move-over behavior between the two EVA alerts (Weibull, Lidestam, & Prytz, 2024). However, based on the results it was not possible to conclude why there was no difference between the alert types. There were differences in modality, language, and the message that was presented. Therefore, it was suggested in Weibull, Lidestam, and Prytz (2024) that forthcoming research should further investigate how modality affects drivers’ move-over behavior.

## False Alerts

A warning must be reliable and ideally presented only when necessary. In addition, there must be enough time for the receiver to prepare for the potential danger. By presenting an alert too early, there is an increased risk of the threat disappearing and therefore causing a false alarm (Parasuraman et al., 1997). When a receiver is faced with a false alert, there is an increased risk that the credibility of the warning system will be damaged, resulting in a decreased compliance rate when the warning is presented again (Breznitz, 1984; Dixon et al., 2007).

Besides the issue of diminished trust, there is also a risk of an increased mental workload and an effect on task performance. If different tasks compete for the same processing resources, it will increase workload and degrade responses (Hancock, 1987). Workload and false alarms have previously been shown to affect psychomotor task performance separately but not in combination. The purpose of an experiment by Bliss and Dunn (2000) was therefore to explore the possible interaction of workload and false alarms on task performance. The purpose was to perform a primary task consisting of a difficult psychomotor task battery three times with an increasing difficulty level. During the task, the participants had to react to an alarm with variable reliability. The participants were assigned to three different groups with either a low, medium, or high alarm presentation rate. The results indicate that increasing the primary task load and alarm task workload degrade alarm response performance. Furthermore, participants would adjust their alarm response frequency to the reliability of the alarm system. However, some participants chose to respond to every alarm, and one participant chose to respond to none. This behavior has been observed in previous research, 103 for instance, Bliss (1993), where 10% of participants chose an all-or-none strategy. When asked about their choice of strategy, the all-or-none participants reported that it was the most effective way to maximize their attention to the primary task (Bliss, 1993). In driving, a greater alarm task workload could result in drivers focusing less on the primary task, that is, driving. Such distraction, especially if unnecessary, is potentially dangerous (LeBlanc et al., 2008). Therefore, it is important to create effective warning systems where the benefits and risks of missed and false alarms are considered. Previous research with false alarms in in-car warnings suggests that a limited number of false alarms does not influence driver actions, but if the false alarms are in the majority, they will affect drivers’ compliance (Ben-Yaacov et al., 2002; Dingus et al., 1997). These studies were performed using forward collision warnings, and in both experiments, the drivers would maintain a larger distance to the vehicles ahead if they had received a majority of false alarms.

There is, to the authors’ knowledge, no available research on possible order effects in false alarms. Order effects in terms of primacy and recency effects are well-studied within psychology. The order in which stimuli is presented is known to affect humans in other contexts, such as the serial-position effect (Ebbinghaus, 1913). However, when it comes to false alarms, there is no available research. First impressions are known to last, and if there is a similar effect in warning compliance, it would be beneficial for designers and human factors engineers to be aware of this possible effect.

The false warning effect is relevant in the EVA warning context because the alerts are based on the most probable route of the emergency vehicle. If, for instance, the driver of the emergency vehicle decides on a new route than what is considered the most probable, there is a risk of drivers receiving an EVA alert, but the emergency vehicle will never show up. The effect of false warnings on drivers’ move-over behavior was explored in Weibull, Lidestam, and Prytz (2024). The study found that false warnings impaired move-over behavior compared to drivers who received only true warnings. However, due to a lack of statistical power, it was not possible to analyze the possible order effects of false and true warnings. Therefore, in the present study, the aim is to further investigate these results by locating differences in how alert modalities and order effects in false alarms affect move-over behavior. The following research questions were formulated:

RQ1: How do auditory and visual modalities for EVA warnings affect move-over behavior?
RQ2: Is there an order effect in false warnings?
RQ3: How do false warnings presented through different modalities affect move-over behavior?

## Method

This research complied with the American Psychological Association Code of Ethics, and written informed consent was provided by each participant.

## Experimental Design

The overall experimental design was 2 (Event: First vs. Second) × 2 (Modality: Auditory vs. Visual) × 2 (Warning combination: True-False-True vs. False-True-True) split-plot factorial design. Modality and Warning combination were between-group variables and Event within-group variable.

### Participants

Most participants were recruited through social media advertisements, while ten were students recruited via flyers distributed on campus. Every participant received cinema tickets worth 120 SEK (∼13 USD) as compensation for their involvement in the study.

Out of 88 recruited participants, six were excluded due to technical issues, and two participants withdrew from the experiment due to simulator sickness, leaving a final sample of 80 (20 women, 60 men). Participants ranged in age from 19 to 85, with a mean age of 53 years (*SD* = 17.6). Participation required a valid driver’s license. Of these, 44% had permits to drive heavier vehicles and trailers, 15% drove professionally, 48% drove daily, 68% had visual corrections and wore glasses, and 15% had hearing impairments. They had, on average, had their driver’s license for 32 years (*SD* = 17.6). The participants reported that they drove between 0–2,000 km/week (*M* = 330, *SD* = 417).

### Materials

**Figure 1.**
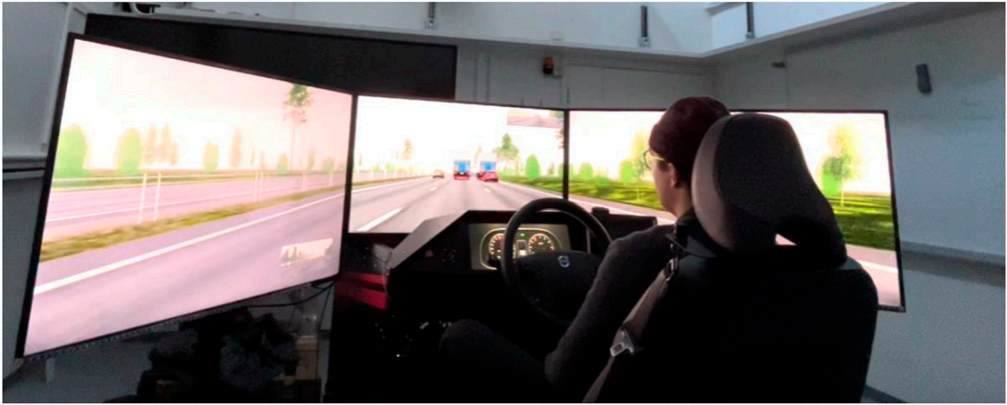
The simulator used in the present study

A fixed-based simulator with three screens was used (Figure 1). The simulator featured three larger monitors, with the left and right screens angled at 45 degrees to provide a realistic peripheral view, including both side-view mirrors. The monitors on the side each measured 55″ diagonally, and the monitor in the center measured 43″.

An auditory and a visual EVA alert were used in the experiment. The auditory alert featured a male voice repeating the phrase in Swedish, “Varning! Utryckande fordon! Var god ge fri väg.” (“Warning! Emergency Vehicle. Please Make Way”), identical to the auditory alert used in the previous study (Weibull, Lidestam, & Prytz, 2024). The same message was displayed in the visual alert. Both alerts were presented approximately 18 seconds before the emergency vehicle passed and continued until it was alongside the driver.

**Figure 2.**
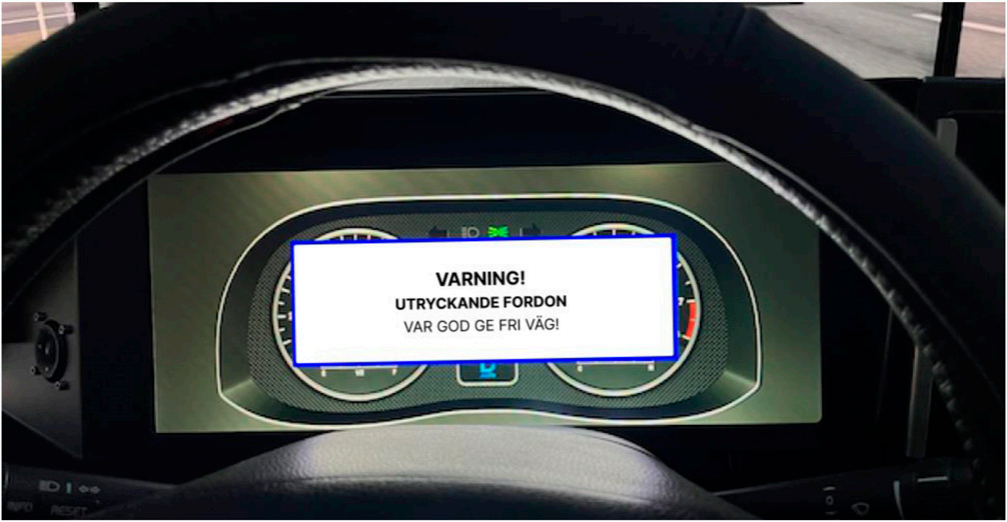
The visual alert used in this experiment, “Warning! Emergency Vehicle. Please Make Way”

To design a visual alert that matches the auditory alert pilot tests were conducted. The goal of the visual alert was not to create an optimal alert, but an alert that matched the auditory alert in terms of urgency, credibility, and clarity. The visual alert was in white and blue colors. Traditional warning colors like red, yellow, and orange were deemed to express a higher level of urgency compared to the urgency expressed by the voice used in the auditory alert. The warning was presented in the instrument cluster (Figure 2).

### Design

The driving scenario took place on a simulated three-lane highway with traffic consisting of cars, vans, and trucks, primarily in the two rightmost lanes to encourage participants to use the left lane. The only vehicles traveling in the left-most lane were one speeding car and the emergency vehicles. The speeding car was included to ensure that not all vehicles approaching the participant in the left-most lane were emergency vehicles.

Each participant received three EVA alerts (visual or auditory), classified as true (emergency vehicle appeared) or false (no emergency vehicle appeared). The sequence was True-False-True (TFT) or False-True-True (FTT), with the final warning always true. By keeping the number of false and true alarms constant but changing the order, the goal was to examine the possible order effects and behavior in the third emergency vehicle event.

Participants (*N* = 80) were divided into four groups based on warning modality and sequence: Auditory (TFT), Visual (TFT), Auditory (FTT), or Visual (FTT). When a warning was true, an emergency vehicle passed by about 18 seconds after the alert was issued, with participants encountering both police cars and ambulances. In the case of a false warning, the EVA warning was presented for 18 seconds and then disappeared without the emergency vehicle showing up. The 18-second alert time was selected based on pilot testing and surveys including 309 participants from five driving simulator experiments with EVA alerts. The conclusion from the surveys was that drivers prefer to be alerted 15–20 seconds before the emergency vehicle is expected to catch up with them (Weibull et al., 2023b).

### Procedure

Participants received study information, signed a consent form, and were briefed on the experiment, which included a demographics questionnaire, a simulation session, and an opinion questionnaire. The participants were told that the experiment would include a novel technical solution but were unaware that it was an EVA alert. Participants were informed about potential simulator sickness and asked whether they had prior experience with simulators or symptoms of simulator sickness.

Before driving in the simulator, participants completed a background survey collecting demographic information, including age, gender, visual and auditory impairments, and driving experience. The participants were briefed on how to drive the simulator and informed that the 28-minute simulation would start at 30 km/h to minimize simulator sickness, gradually increasing to 120 km/h with cruise control. They were instructed to maintain 120 km/h, and that they could brake if needed. The simulation began with participants driving alone on a highway. After driving for 11 minutes, the participant was approached by an ambulance from behind. After an additional 8 and 16 minutes, the same event would occur again. The ambulance was driving at 165 km/h, which was the same speed used in Weibull, Lidestam, and Prytz (2024). After the simulation ended, the participants completed a survey about their experiences. The survey assessed the perceived impact of the auditory or visual warning they received while driving. Both surveys included yes/no questions and 7-point Likert scale items. After filling out the post experiment questionnaire, they were informed about the purpose of the study and received a gift card to thank them for their participation.

## Results

An emergency vehicle delay measure was used to examine how quickly the drivers moved over when receiving the EVA alerts, true and false. The delay was calculated by comparing the distance the emergency vehicle would have traveled at its preferred speed with how far it actually traveled if the participant’s vehicle blocked its path. The difference in distance was converted into how many seconds the emergency vehicle was delayed. The delay was thus zero if the participant moved over in time so that the emergency vehicle could maintain its preferred speed.

To examine the effects of modality and false warnings, a 2 (Event) × 2 (Modality) × 2 (Warning combination) repeated measures ANOVA was performed. Data points at least three times the interquartile range were considered outliers and removed. A total of eight out of 160 event measurements were excluded. The threshold of statistical significance was set to α = 0.05. Paired *T* tests with Bonferroni correction were used for post hoc analysis.

### Modality

There was a significant effect of modality such that the drivers who received an auditory alert (*M* = 3.37 *s*, *SE* = 0.59 *s*) caused less delay for the emergency vehicle, compared to the drivers who received a visual alert (*M* = 5.41 *s*, *SE* = 0.63 *s*), F(1,68) = 5.61, *p* = .02, $\eta_{p}^{2}$ = 0.08.

### Order of False Warning

When comparing the delay measurements between the two orders of false warnings, the TFT group (*M* = 2.58 *s*, *SE* = 0.60 *s*) had a shorter delay time than the FTT group (*M* = 6.20 *s*, *SE* = 0.62 *s*). The difference was statistically significant, F(1,68) = 17.68, *p* < .001, $\eta_{p}^{2}$ = 0.21.

### Event

There was no significant difference in delay between the first (*M* = 5.01 *s*, *SE* = 0.55 *s*) and second (*M* = 3.78 *s*, *SE* = 0.62 *s*) true interaction with the emergency vehicle, F(1,68) = 2.38, *p* = .13.

### Modality × Order of False Warning

There was no significant interaction effect of modality and order of false warning, F(1,68) = 0.83, *p* = .37.

### Modality × Event

There was no significant interaction effect of modality and event, F(1,68) = 2.75, *p* = .10.

### Event × Order of False Warning

**Figure 3.**
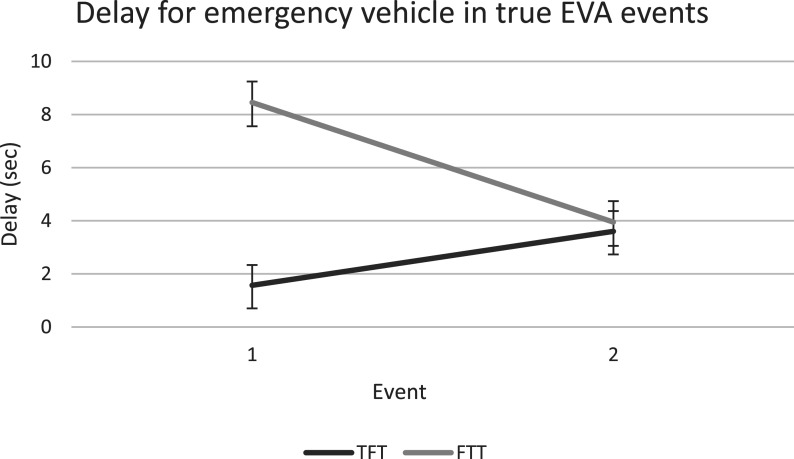
Comparison of the mean delay time (±SE) for the different warning combinations in the true emergency vehicle events

There was a significant interaction effect of the event and the order of the false warning, F(1,68) = 16.54, *p* = <.001, $\eta_{p}^{2}$ = 0.20 (Figure 3). In the first true event, drivers whose first alert was true (TFT) (*M* = 1.58 *s*, *SE* = 0.76 *s*) had a significantly shorter delay time than the drivers who, when receiving their first true EVA alert had previously experienced a false alert (FTT) (*M* = 8.45 *s*, *SE* = 0.79 *s*). Among the drivers who received an FTT warning combination, there was a significant decrease in delay time between their first (*M* = 8.45 *s*, *SE* = 0.79 *s*) and second true interactions (*M* = 3.95 *s*, *SE* = 0.90 *s*). The same effect was not observed in the TFT warning combination group (First true event: *M* = 1.57 *s*, *SE* = 0.76 *s*; Second true event: *M* = 3.6 *s*, *SE* = 0.87 *s*).

### Modality × Order of False Warning × Event

There was no significant effect of modality, order of false warning, and event, F(1,68) = 2.82, *p* = .10.

## Discussion

Aligned with previous research (Bella & Silvestri, 2017; Huang et al., 2024; Lylykangas et al., 2016), there was a significant effect between the different warning modalities. The drivers moved over more quickly for the approaching emergency vehicle when they received an auditory EVA alert compared to a visual EVA alert. This finding supports the idea that in-car alerts should be auditory so that drivers are not visually distracted. However, one potential issue with auditory warnings is the risk of the driver not hearing the alert. This could, for instance, be due to noisy passengers in the car or hearing impairments. A dual or multi-modality alternative was not explored in this study but might be favorable in scenarios where the driver must perceive the warning in time because they are associated with shorter response times (Wang et al., 2022). EVA alerts, however, are possibly not a type of alert where multimodality should be used. Instead, it should be reserved for when a more urgent response is needed, such as a forward collision warning.

Unlike in Weibull, Lidestam, and Prytz (2024), there was no significant main effect of Event. In Weibull, Lidestam, and Prytz (2024), the participants were quicker at moving over in the later events compared to the first emergency vehicle event. One reason why there was no significant effect in the current study is the differences in the dependent variables used for analysis. In Weibull, Lidestam, and Prytz (2024), a measurement of “move over time” was used instead of delay time, as was used in the current study. The dependent variable “move over time” was a more sensitive measurement to find differences between the events compared to delay time. Therefore, it is possible that if analyzed using move over time, the decrease between the first and second event would be significant in the current study, similar to Weibull, Lidestam, and Prytz (2024).

The results suggest an effect of false alarms. The drivers who had a TFT warning combination had an overall shorter delay time compared to the drivers who received the warning combination FTT. When comparing the true emergency vehicle events separately, there was a significant effect in the first true event but not in the second (TFT vs. FTT). This suggests that drivers were more affected if they had experienced a false warning previously, compared to the drivers who experienced a true alarm first. However, in the second true interaction, there was no significant difference between the two order groups. This suggests that the order effect in false alarms might not be that strong, but instead, it is more important to consider the proportion of false alarms. This is aligned with the findings of Ben-Yaacov et al. (2002) and Dingus et al. (1997), where it was suggested that false alert will affect driver behavior when they are in the majority compared to true alarms.

False alarms are important to consider in relation to trust. When using driving support systems, drivers must align their expectations with the system’s functionality. If the user trusts the system too much, it can lead to overdependence, resulting in scenarios where the user relies on the system without confirmation—such as failing to monitor the road while using autopilot features. Moreover, research has shown that drivers tend to place too much trust in Adaptive Cruise Control, which can diminish their attention during critical safety situations (Larue & Rakotonirainy, 2022). Conversely, if a user is overly critical of the system’s abilities, it may prevent them from utilizing a feature that could be advantageous. For example, a driver might not believe that Adaptive Cruise Control will apply the brakes effectively in time. It is therefore important to communicate a level of historical system reliability to the driver (Lee & See, 2004). In the current experiment, the participants were not told about the reliance of the EVA system. For future experiments, it might be a wise idea to be transparent about the reliability of the system so that an appropriate level of trust can be established.

The C-HIP model can be used to understand how humans receive and judge safety information (Wogalter, 2006). For instance, how the channel (Modality) may play a role. However, in its current form the C-HIP model does not allow for understanding of the communication process of false alarms. The model assumes that there is a threat that must be communicated to the receiver. In the case of a false alarm, the warning can effectively be communicated to the receiver but there is no need for the warning to be communicated, for example, no approaching emergency vehicle. This discrepancy cannot be analyzed in the C-HIP model in its current form.

One limitation of the current study is that each participant contributed to only three delay measurements. The reason behind this design was the increased risk of fatigue if the driving scenario was prolonged. The participants drove in the simulator for 30 minutes on a straight highway. Several of the participants complained of boredom after they had been driving for 20 minutes. Therefore, it was not an option to increase the time of the driving scenario. Another way to increase the number of data points per participant would have been to decrease the time between the interactions. This would have increased the statistical power of the study and decreased the risk of Type II errors. However, if the time between events had been shortened there would be a risk of drivers remaining too alerted. Allowing time between the events increased the ecological validity of the experiment.

Future research may consider the possible effects of driver age. Even though Weibull et al. (2023) did not see an age effect, there are other studies that suggest that a driver’s age could affect driver behavior. For instance, older drivers are more prone to mind-wandering (Feng et al., 2017) and are less affected by a high false warning rate compared to younger drivers (Dingus et al., 1997).

The findings further support that EVA warnings can assist drivers in interactions with emergency vehicles. False warnings and modality are important aspects to consider when designing EVA warnings.

## Conclusion

The drivers who had previously experienced a false alarm were slower at moving over when faced with a true alarm, compared to drivers who first received a true alarm. The experience of a false alarm affects drivers’ willingness to move over. However, there was no significant difference in move-over behavior once the drivers had received an equal number of true and false alarms. This suggests that drivers are robust in handling false alarms if they have experienced a true alarm previously. Furthermore, aligned with previous research, drivers who received an auditory warning were quicker at moving over compared to drivers who received a visual warning.

## Key Points

• False alarms affect move-over behavior.
• Drivers were less affected by a false alarm if they had received a true alarm before
• After receiving an equal number of false and true alarms, drivers were equally quick at moving over, despite the order of alarms.
• Drivers who received an auditory warning moved over more quickly compared to drivers who received a visual warning.
